# Combined small RNA and gene expression analysis revealed roles of miRNAs in maize response to rice black-streaked dwarf virus infection

**DOI:** 10.1038/s41598-018-31919-z

**Published:** 2018-09-10

**Authors:** Aiqin Li, Guanghui Li, Yuhan Zhao, Zhaodong Meng, Meng Zhao, Changsheng Li, Ye Zhang, Pengcheng Li, Chang-Le Ma, Han Xia, Shuzhen Zhao, Lei Hou, Chuanzhi Zhao, Xingjun Wang

**Affiliations:** 1 Biotechnology Research Center, Shandong Academy of Agricultural Sciences; Shandong Provincial Key Laboratory of Crop Genetic Improvement, Ecology and Physiology, Jinan, 250100 PR China; 2 College of Life Sciences, Shandong Normal University, Jinan, 250014 PR China; 3 Maize Research Institute, Shandong Academy of Agricultural Sciences, Jinan, 250100 PR China

## Abstract

Maize rough dwarf disease, caused by rice black-streaked dwarf virus (RBSDV), is a devastating disease in maize (*Zea mays* L.). MicroRNAs (miRNAs) are known to play critical roles in regulation of plant growth, development, and adaptation to abiotic and biotic stresses. To elucidate the roles of miRNAs in the regulation of maize in response to RBSDV, we employed high-throughput sequencing technology to analyze the miRNAome and transcriptome following RBSDV infection. A total of 76 known miRNAs, 226 potential novel miRNAs and 351 target genes were identified. Our dataset showed that the expression patterns of 81 miRNAs changed dramatically in response to RBSDV infection. Transcriptome analysis showed that 453 genes were differentially expressed after RBSDV infection. GO, COG and KEGG analysis results demonstrated that genes involved with photosynthesis and metabolism were significantly enriched. In addition, twelve miRNA-mRNA interaction pairs were identified, and six of them were likely to play significant roles in maize response to RBSDV. This study provided valuable information for understanding the molecular mechanism of maize disease resistance, and could be useful in method development to protect maize against RBSDV.

## Introduction

Maize is one of the most important and widely distributed crops in the world, providing more than a billion tons of human food and animal feed every year (FAO, http://faostat.fao.org/). However, maize production is threaten by a number of diseases, including maize rough dwarf disease (MRDD). MRDD is a devastating disease for maize, resulting in severe growth abnormalities, such as plant dwarfing, dark green leaf and a vein clearing. In China, the pathogen of MRDD is *Rice black-streaked dwarf virus* (RBSDV), a *Fijivirus* in the family of *Reoviridae*^1^. Recently, the disastrous losses caused by RBSDV have already spread into most maize growing districts of China^2^. Although some maize germplasm displayed low level of resistance to RBSDV, the high resistant varieties were rare. The control of MRDD mainly depends on cultivation management to avoid small brown plant hoppers (BPHs; *Laodelphax striatellus*), which transmitted the virus to maize and rice. BPHs are a class of long-distance migratory pest and difficult to control. Therefore, improving maize resistance to RBSDV and planting resistant cultivars are of great necessity. To increasing the resistance of maize, understanding the molecular mechanism of RBSDV pathogenesis is highly required.

During the long history of evolution, plants have evolved a series of flexible defense responses to resist the invasion of pathogenic microorganisms. Hypersensitive response (HR) and systemic acquired resistance (SAR) were two important responses, which usually happened in infection local tissues and uninfected tissues, respectively^3^. In the defense responses, a large number of genes, such as the defense-related genes, pathogenesis-related (PR)-protein genes could be induced. For example, the expression of *PR-1*, *PR-2* and *PR-5* were induced for increased resistance against *Pernospore parasitica* and *Pseudomonas syringae* in *Arabidopsis*^4,5^. Other genes, such as p450 monooxegenases, hypersensitivity-related genes, cellulases, ABC transporters, receptor-like kinases, serine/threonine kinases, phosphoribosylanthranilate transferases and hypothetical R genes, were induced upon taxonomically distinct tobacco rattle virus (TRV) infection^6^. Gene expression profile of RBSDV-infected maize was investigated using microarray, and the results demonstrated that the expressions of various resistance-related genes, cell wall and development related genes were altered^7^. These results provided valuable information to uncover the molecular mechanisms to understand symptom development in rough dwarf-related diseases. Recently, studies demonstrated that pathogen infection not only change the expression of disease resistance genes but also the endogenous miRNAs^8–11^.

MiRNA, is a member of endogenous and non-coding small RNA with the length of 20–24 nt. MiRNA negatively regulate gene expression via mRNA cleavage or translational inhibition of its targets, exhibiting important roles in regulation of plant growth, development, and adaptation to stresses^12–15^. Numerous miRNAs have been reported to be induced by pathogen infection and contribute to the gene expression reprogramming in host defense responses. Based on deep sequencing data and RNA-blot assay, a group of known rice miRNAs were differential expressed upon *Magnaporthe oryzae* infection. Overexpression of miR160a and miR398b enhanced disease resistance in the transgenic rice^16^. Induction of miRNAs were also observed in wheat and peanut after infected with powdery mildew and bacterial wilt pathogens, respectively^9,11^. In tomato, a member of NBS-LRR disease resistance (R) gene were proved to be regulated by miR482 and miR2118^17^. Studies revealed that miR472 and RDR-mediated silencing pathway represented a key regulatory checkpoint modulating both PTI (pathogens induce pathogen-associated molecular pattern (PAMP)-triggered immunity) and ETI (effector-triggered immunity) via post-transcriptional control of R genes^18^. Based on microarray data, the expression of 14 stress-regulated rice miRNAs was induced by southern rice black-streaked dwarf virus (SRBSDV) infection^10^.

Up to now, a total of 321 maize miRNA mature and 172 precursors sequences have been deposited in miRBase (www.mirbase.org). Many miRNAs have been confirmed to play regulation roles in maize growth, development and stress response^19–22^. For example, maize *ts4* encodes a member of miR172, controls sex determination and meristem cell fate by targeting Tasselseed6/indeterminate spikelet1, an APETALA2 floral homeotic transcription factor^23^. Four maize miRNAs, miR811, miR829, miR845 and miR408, were differentially expressed in response to *Exserohilum turcicum*, a major pathogenic fungus of maize causing northern leaf blight. Over-expression of miR811 and miR829 conferred transgenic lines with high degree of resistance to *E*. *turcicum*^8^. However, there is no report about miRNA response in maize upon RBSDV infection.

In this study, we employed high-throughput sequencing technology to characterize the changes in transcriptome and miRNAome following RBSDV infection. Integrated analysis of gene and miRNA datasets revealed miRNA-mRNA interaction pairs that involved in leaf development, cell wall synthesis and degradation, plant-pathogen interaction. Our results provided valuable information to reveal the molecular mechanisms between the interaction of RBSDV and maize.

## Results

### Small RNA deep sequencing and data analysis

**Figure 1:**
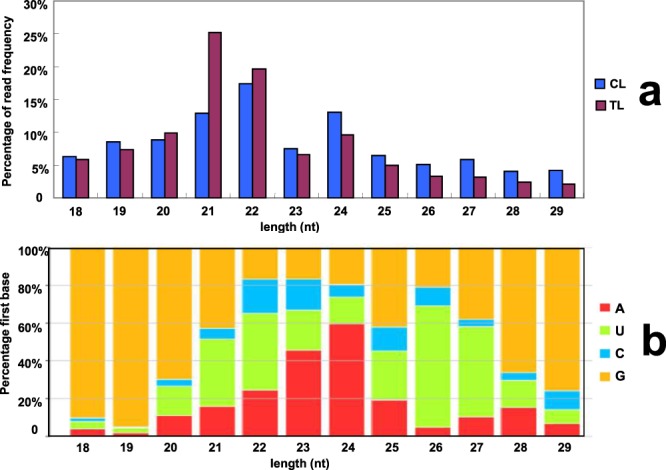
Statistics of length distribution and first nucleotide bias of small RNA libraries. a: Length distribution of small RNAs identified from CL and TL libraries, b: First nucleotide bias analysis of total small RNAs.

Maize B73 was naturally infected by RBSDV in the field where the Maize Rough Dwarf Disease happened seriously. The control plants were grown in the field and covered by net to prevent the planthoppers, the carrier of the virus. To test whether plants were infected by RBSDV, two pair of primers pS6–604 and pS7–342 were designed according to the genome sequences of segment S6 (GenBank No: HF955010) and segment S7 (GenBank No: HF955011) of RBSDV, respectively. These two pair of primers were used to amplify in the maize individuals with RBSDV symptoms. As a result, the virus genes were amplified in all treatment plants, and could not be detected in control plants (Supplementary Fig. S1). These results suggested that the phenotype/symptoms were caused by RBSDV. To identify small RNAs from maize, two libraries generated using RBSDV infected plants (TL1 and TL2) and two libraries (CL1 and CL2) generated using the control plants were constructed for high-throughput sequencing. A total of 23,056,821, 20,003,963, 26,678,964 and 20,585,338 raw reads were obtained from CL1, CL2, TL1 and TL2, respectively (Supplementary Table S1). After removing low quality reads, reads less than 18 nt and reads longer than 29 nt, a total of 9,274,931, 8,351,418, 9,552,470 and 13,037,763 clean reads remained from CL1, CL2, TL1 and TL2 libraries, respectively (Supplementary Table S1). These clean reads were used for further analysis. Firstly, clean reads were aligned with maize genome (B73 RefGen_V2, release 5b.60) and Rfam database. Reads annotated as rRNA, snRNA (small nuclear RNAs), snoRNA (nucleolar RNAs), repbase (reads positioned at repeat loci) and tRNA were identified (Supplementary Table S2). The distribution of small RNAs identified from CL and TL libraries is summarized (Fig. 1a). It was also shown that 21-nt small RNAs were the predominant class in maize, followed by 22-nt and 24-nt small RNAs. After infected with RBSDV, the number of 20-, 21- and 22-nt small RNAs in TL libraries increased, while the number of other small RNAs decreased compared with CL libraries. The first nucleotide bias of small RNAs was analyzed. For the small RNAs of 20–22 nt, the canonical length of miRNAs, a strong bias for U of the first nucleotide was observed (Fig. 1b). The small RNA sequencing data has been deposited in NCBI Short Read Archive (SRA) database (BioProject ID: PRJNA438075, Accession number: SRR6829172-SRR6829175).

### Identification of known and novel miRNAs in maize

Comparison the maize small RNAs to the miRBase allowed us to identify 76 known miRNAs, belonging to 26 miRNA families (Supplementary Table S3). Previous studies found that there were twenty miRNAs families were conserved in *Arabidopsis*, *Oryza ostiva* and *Populus trichocarpa*^13,24^. In our dataset, nineteen conserved miRNA families were detected in maize. In addition, seven known but non-conserved miRNA families including MIR408, MIR528, MIR529, MIR827, MIR1432, MIR2118 and MIR2275 were also identified. The normalized expression level of miR166f was 103,108 (TP10M, Numbers of tags per ten million), representing the most abundant miRNAs. The abundance of miR395o-5p, miR2118d, miR395k-3p, miR167g-3p, miR167c-3p, miR408b-3p, miR169r-3p, miR2275a-5p, miR398b-3p and miR398a-3p was low in both sRNA libraries (Supplementary Table S3).

According to the criteria as described in previously^25^, a total of 226 potential novel miRNAs were identified. The length of novel miRNAs ranged from 20 to 22 nt, and more than 93% novel miRNAs with the length of 21–22 nt (Supplementary Table S4). The negative folding free energies of these precursors hairpin structures ranged from −89.8 to −16.1 (kcal/mol) with an average of −44.08 kcal/mol, which is similar to the results from other plants. Some of these novel miRNAs were specifically detected in control or treatment libraries. For example, zma-miRn177 was detected only in control libraries, while zma-miRn223 and zma-miRn224 were observed only in treatment libraries.

### Target prediction of maize miRNAs and function annotation

**Figure 2:**
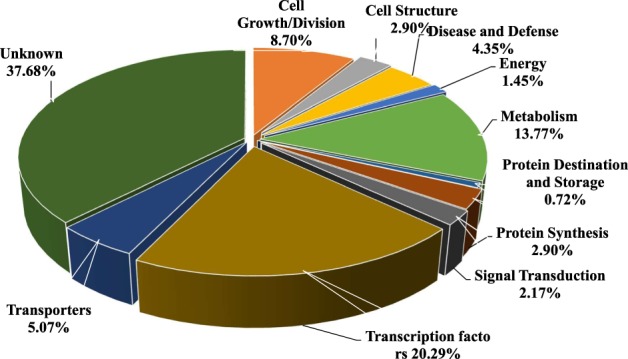
Function classification of the target genes of novel miRNAs.

To gain a better understanding of the regulation roles of maize known and novel miRNAs, target genes were predicted using psRNATarget software by comparing miRNA sequences against maize B73 reference genome. A total of 213 targets of 50 known miRNAs and 138 targets of 75 novel miRNA candidates were identified (Supplementary Table S5-S6). Functional annotation of these target genes showed that many defense related genes were regulated by known miRNAs. For example, peroxidase 2 gene (GRMZM2G427815), LRR receptor-like serine/threonine-protein kinase gene (GRMZM2G304745), and a Zea mays rust resistance protein rp3–1 (GRMZM2G045955) were targeted by zma-miR399a-5p, zma-miR390b-5p and zma-miR528b-5p, respectively (Supplementary Table S5). For the targets of novel miRNAs, 37.68% of them were genes with unknown function and 20% of them were transcription factors. Our data showed that many defense related genes were also regulated by novel miRNAs including a plant viral-response family protein gene (GRMZM2G171036), which was regulated by miRn200 (Fig. 2, Supplementary Table S6).

### MiRNA expression profiles upon RBSDV infection

**Figure 3:**
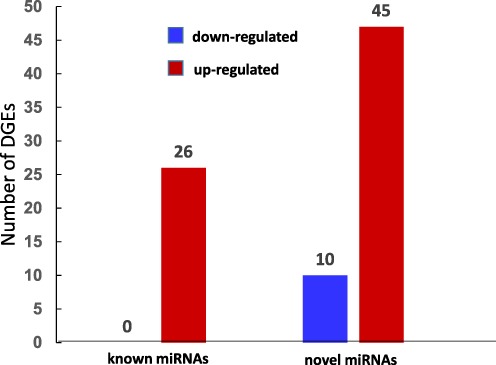
Number of different expressed miRNAs in response to RBSDV.

**Figure 4:**
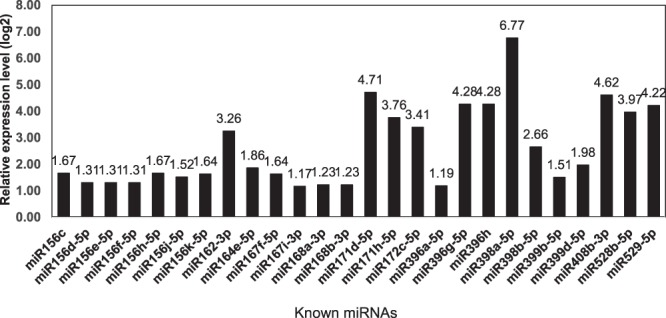
Different expressed known miRNAs identified in sRNA libraries.

To analyze the expression change of miRNAs in response to RBSDV, the abundance of miRNAs was normalized using numbers of tags per ten million (TP10M), and the relatively expression level of miRNA was calculating by log_2_Ratio (TL/CL). A total of 81 differentially expressed miRNAs (|log_2_| ≥ 1.0) were identified, including 26 known miRNAs and 55 novel miRNAs (Fig. 3). Interestingly, the 26 differential expressed known miRNAs were all up-regulated, and the overall expression levels of all known miRNAs showed up-regulation trend in response to RBSDV (Fig. 4, Supplementary Table S7). Among the 55 differential expressed novel miRNAs, 45 were up-regulated and 10 were down-regulated (Fig. 3). MiRn177 was expressed only in control samples, miRn223 and miRn224 expressed only in virus treated samples. In addition, our results showed that the expression levels of four novel miRNAs, miRn222, miRn225, miRn226 and miRn146 were significantly increased after infected with RBSDV (Supplementary Fig. S2, Supplementary Table S4).

### Global mRNA expression profiles in maize in response to virus infection

In order to identify the global gene expression alteration upon virus infection, we used the next generation sequencing technology to analyze the transcriptome of maize before and after virus infection. A total of 8.13 Gb data was generated, comprised of more than 40 million reads (Supplementary Table S8). Sequencing randomness analysis was tested for estimating the gene whether or not random distributed in different positions on each genes. The statistical analysis result showed that the sequencing in all samples was in good randomness (Supplementary Fig. S3a). Saturation analysis showed that the number of genes increased with the total number of tags and reached a plateau after 2.5 million tags (Supplementary Fig. S3b). Using TopHat alignment, more than 89% of the reads could be successfully mapped to B73 genome, which covers 27,554 genes. The RNA-seq data have been deposited at SRA database (BioProject ID: PRJNA438075, Accession number: SRR6829168-SRR6829171).

### Identification and function analysis of differentially expressed genes (DEGs)

**Figure 5:**
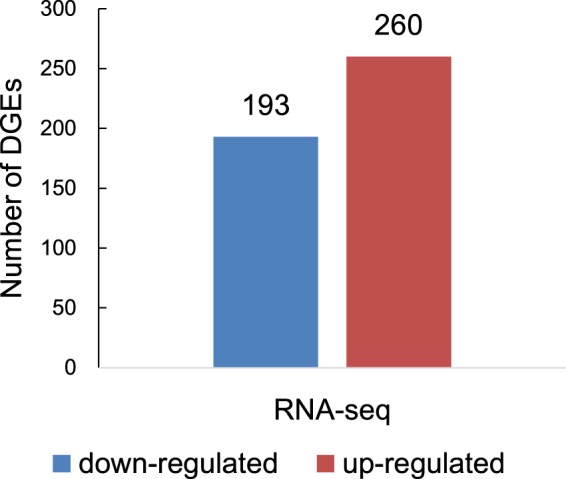
Different expressed genes in maize response to RBSDV.

To identify differential expressed genes (DEGs) response to virus infection, comparison analysis between control and treated transcriptome libraries was performed. The expression level of genes were normalized by FPKM (expected number of fragments per kilobase of transcript sequence per millions base pairs sequenced). The pearson correlation values between two control (E1 and E3) and two treatment libraries (E2 and E4) were 0.964 and 0.948, respectively (Supplementary Fig. S3c,d). Under the criterion of P-value ≤ 0.001 and |log_2_| ≥ 1.0, a total of 453 DEGs were found, including 260 up-regulated and 193 down-regulated genes (Fig. 5). The function of these genes were annotated by alignment with Nr and SWISSPROT Database (Supplementary Table S9). Functional annotation indicated that many disease resistance related genes were up-regulated after RBSDV infection. For example, glutathione S-transferase, lipoxygenase, lectin-like receptor protein kinase, O-methyltransferase 8, pathogenesis-related protein 10 and non-specific lipid-transfer protein, etc. (Supplementary Table S9).

In order to explore the functions of these differential expressed genes that are responsive to RBSDV infection, Gene ontology (GO), COG annotation and Pathway enrichment analysis were performed. From our dataset, 168 of 453 differential expressed genes have significant homologies in COG database and were assigned into 25 categories (Supplementary Table S9, Supplementary Fig. S4). Among them, “General function prediction only”, “Carbohydrate transport and metabolism”, “Replication, recombination and repair”, “Amino acid transport and metabolism” and “Energy production and conversion” ranked the top five categories (Supplementary Table S9, Supplementary Fig. S4). To further understand the metabolic and regulatory process for RBSDV-responsive, all up- and down-regulated genes were subjected to BGI WEGO program for GO analysis. The detailed summary of GO classification showed that cell, cell part, organelle were the most abundant ones in cell component categories. About molecular function category, the most abundant were binding and catalytic. The last category was biological process, in which cellular process, metabolic process, and response to stimulus were enriched (Supplementary Fig. S5).

According to KEGG analysis, 77 DEGs were annotated into 65 pathways. Among them, 23 pathways were enriched in response to RBSDV infection including two photosynthesis related pathways, photosynthesis and carbon fixation in photosynthetic organisms (Table 1). In addition, many DEGs were enriched in pathways involved in metabolite or secondary metabolite synthesis, such as starch and sucrose metabolism, pyruvate metabolism, butanoate metabolism, alanine, beta-Alanine metabolism, glutathione metabolism, aspartate and glutamate metabolism, suggesting significant metabolic changes after RBSDV infection. We found four genes, including a putative coronatine-insensitive protein (GRMZM2G035314, log_2_ = 2.16), a respiratory burst oxidase-like protein (GRMZM2G043435, log_2_ = 1.07), a heat shock protein HSP82 (GRMZM5G833699, log_2_ = 1.78) and one unknown protein (GRMZM2G151519, log_2_ = 1.44), were all enriched in plant-pathogen interaction pathway. Interestingly, these genes were all up-regulated in response to virus infection (Supplementary Table S9).

**Table 1:** Enriched pathways in maize response to RBSDV.

Number	Pathways	DEGs with pathway annotation (77)	All genes with pathway annotation (4137)	P-value	Pathway ID
1	Photosynthesis	8 (10.39%)	124 (3%)	1.93E-03	ko00195
2	Carbon fixation in photosynthetic organisms	7 (9.09%)	97 (2.34%)	1.97E-03	ko00710
3	Starch and sucrose metabolism	7 (9.09%)	135 (3.26%)	1.21E-02	ko00500
4	Pyruvate metabolism	5 (6.49%)	85 (2.05%)	2.02E-02	ko00620
5	Alanine, aspartate and glutamate metabolism	4 (5.19%)	57 (1.38%)	2.08E-02	ko00250
6	Butanoate metabolism	3 (3.9%)	32 (0.77%)	2.09E-02	ko00650
7	Pentose phosphate pathway	3 (3.9%)	52 (1.26%)	7.16E-02	ko00030
8	Cysteine and methionine metabolism	4 (5.19%)	85 (2.05%)	7.25E-02	ko00270
9	Glyoxylate and dicarboxylate metabolism	3 (3.9%)	55 (1.33%)	8.17E-02	ko00630
10	Ascorbate and aldarate metabolism	2 (2.6%)	27 (0.65%)	8.90E-02	ko00053
11	beta-Alanine metabolism	2 (2.6%)	28 (0.68%)	9.47E-02	ko00410
12	Glycolysis/Gluconeogenesis	5 (6.49%)	135 (3.26%)	1.05E-01	ko00010
13	Glutathione metabolism	3 (3.9%)	67 (1.62%)	1.28E-01	ko00480
14	Phenylpropanoid biosynthesis	3 (3.9%)	71 (1.72%)	1.45E-01	ko00940
15	Fructose and mannose metabolism	3 (3.9%)	72 (1.74%)	1.49E-01	ko00051
16	Limonene and pinene degradation	1 (1.3%)	9 (0.22%)	1.56E-01	ko00903
17	ABC transporters	1 (1.3%)	9 (0.22%)	1.56E-01	ko02010
18	Steroid biosynthesis	2 (2.6%)	39 (0.94%)	1.63E-01	ko00100
19	Valine, leucine and isoleucine biosynthesis	2 (2.6%)	40 (0.97%)	1.70E-01	ko00290
20	Stilbenoid, diarylheptanoid and gingerol biosynthesis	1 (1.3%)	10 (0.24%)	1.71E-01	ko00945
21	Fatty acid biosynthesis	2 (2.6%)	41 (0.99%)	1.77E-01	ko00061
22	Pentose and glucuronate interconversions	2 (2.6%)	46 (1.11%)	2.11E-01	ko00040
23	Plant-pathogen interaction	4 (5.19%)	129 (3.12%)	2.18E-01	ko04626

### The expression of defense related genes response to RBSDV infection

Previous research has demonstrated that the expression of some defense response-related genes changed significantly when plants were suffered with biotic or abiotic stresses^7,26,27^. In this study, we investigated the expression changes of defense related genes, and found the expression of 90 defense related genes altered after RBSDV infection, including 53 receptor-like protein kinase genes, six WRKY DNA-binding protein genes, two NBS-LRR family genes, nine pathogenesis-related genes, 13 glutathione S-transferase genes, three peroxidase genes, one heat shock protein gene, one ferredoxin and two other disease resistance analog genes (Table 2). Receptor-like protein kinase was an important signal introduction factor involved in plant disease resistance^28^. As shown in Table 2, more than half of these defense related genes encoded diverse receptor-like protein kinases. Among them, the expression of 39 receptor-like protein kinase were up-regulated, while 14 of them were down-regulated upon virus infection. We found that the expression of some of these genes were altered dramatically, for example, the glutathione S-transferase gene (GRMZM2G146913) was up-regulated for about 30 times (log_2_ = 4.9274), the peroxidase gene (GRMZM2G313184) was up-regulated for about 24 times (log_2_ = 4.6027). These results provided important information for us to understand the mechanism under miRNA regulation of disease resistance in maize.

**Table 2:** Differentially expressed defense related genes in response to RBSDV.

Gene ID	Annotation	log2(TL/CL)
**Receptor-like protein kinase**		
GRMZM2G576752	WAK family receptor-like protein kinase	−1.0290
GRMZM2G420882	S-locus receptor-like protein kinase	1.2255
GRMZM2G063533	Serine/threonine-protein kinase NAK	0.8165
GRMZM5G897958	Receptor-like protein kinase HERK 1	0.9184
GRMZM2G006080	Receptor-like protein kinase	0.8098
GRMZM2G152901	Receptor-like protein kinase	1.0048
GRMZM2G162702	Receptor-like protein kinase	2.0344
GRMZM2G391794	Receptor-like protein kinase	1.3456
GRMZM2G034855	Receptor-like protein kinase	0.7051
GRMZM2G081957	Receptor-like protein kinase	−1.1485
GRMZM2G004207	Receptor-like protein kinase	1.2574
GRMZM2G426917	Receptor-like protein kinase	1.1497
GRMZM5G806108	Receptor-like protein kinase	1.3281
GRMZM2G110968	Receptor-like protein kinase	1.8770
GRMZM2G020158	Protein kinase superfamily protein	−1.5095
GRMZM2G473511	Protein kinase superfamily protein	−1.0664
GRMZM2G395778	Protein kinase superfamily protein	−0.7861
GRMZM2G068316	Proline-rich receptor-like protein kinase PERK9	−1.2666
GRMZM2G428964	Proline-rich receptor-like protein kinase PERK8	−0.9519
GRMZM5G832452	Proline-rich receptor-like protein kinase PERK4	−1.1054
GRMZM5G838420	Proline-rich receptor-like protein kinase PERK2	1.2050
GRMZM2G055119	Proline-rich receptor-like protein kinase PERK10	1.6598
GRMZM2G358365	Proline-rich receptor-like protein kinase	3.2080
AC202877.3_FG002	Proline-rich receptor-like protein kinase	0.9396
GRMZM5G872442	Proline-rich receptor-like protein kinase	0.9015
GRMZM2G024024	LysM-domain receptor-like protein kinase	1.9487
GRMZM2G438007	Leucine-rich repeat receptor-like protein kinase	0.9889
GRMZM2G011806	Leucine-rich repeat receptor-like protein kinase	1.3479
GRMZM2G162829	Leucine-rich repeat receptor-like protein kinase	2.0528
GRMZM2G009995	Leucine-rich repeat receptor-like protein kinase	2.1007
GRMZM2G150448	Leucine-rich repeat receptor-like protein kinase	0.9893
GRMZM2G048294	Leucine-rich repeat receptor-like protein kinase	0.8271
GRMZM2G100234	Leucine-rich repeat receptor-like protein kinase	1.0276
GRMZM2G176206	Leucine-rich repeat receptor-like protein kinase	0.7631
GRMZM2G056903	Leucine-rich repeat receptor-like protein kinase	2.0459
GRMZM2G360219	Leucine-rich repeat receptor-like protein kinase	2.6016
GRMZM2G178753	Leucine-rich repeat receptor-like protein kinase	−1.4082
GRMZM2G104384	Leucine-rich repeat receptor-like protein kinase	−0.8126
GRMZM2G082191	Leucine-rich repeat receptor-like protein kinase	0.9897
GRMZM2G168917	Leucine-rich repeat receptor protein kinase EXS precursor	1.8846
GRMZM2G123450	Leucine-rich repeat receptor protein kinase	−1.4308
GRMZM2G377199	Lectin-domain receptor-like protein	−1.1165
GRMZM2G017522	Cysteine-rich receptor-like protein kinase 42	1.8142
GRMZM2G087625	Cysteine-rich receptor-like protein kinase 25	1.2029
GRMZM2G338161	Cysteine-rich receptor-like protein kinase 2	−1.2323
GRMZM2G419318	Cysteine-rich receptor-like protein kinase	1.4175
GRMZM2G009506	Cysteine-rich receptor-like protein kinase	1.6376
GRMZM2G352858	Cysteine-rich receptor-like protein kinase	1.2474
GRMZM2G054023	Lectin-like receptor protein kinase family protein	0.8618
GRMZM2G400694	Lectin-like receptor protein kinase family protein	−1.1888
GRMZM2G142037	Lectin-like receptor protein kinase family protein	2.8308
GRMZM2G400725	Lectin-like receptor protein kinase family protein	0.8367
GRMZM2G089819	Brassinosteroid LRR receptor kinase precursor	1.4135
**WRKY DNA-binding**		
GRMZM2G411766	WRKY DNA-binding domain superfamily protein	−0.6117
GRMZM2G149683	WRKY DNA-binding domain superfamily protein	−1.6621
GRMZM5G851490	WRKY DNA-binding domain superfamily protein	0.9215
GRMZM2G377217	WRKY DNA-binding domain superfamily protein	−1.7191
GRMZM2G004060	WRKY DNA-binding domain superfamily protein	1.2294
GRMZM2G060918	WRKY DNA-binding domain superfamily protein	2.1868
**NBS-LRR disease resistance gene**		
GRMZM2G005452	NBS-LRR type disease resistance protein	0.7430
GRMZM2G092286	TIR-NBS-LRR type disease resistance protein	0.6914
**Pathogenesis-related**		
GRMZM2G156857	Pathogenesis-related	2.2732
GRMZM2G474326	Ethylene-responsive transcription factor 2	−0.9431
GRMZM2G008406	Pathogenesis-related protein PR-1 precursor	1.1899
GRMZM2G112538	Pathogenesis-related protein 10	2.8317
GRMZM2G091742	Pathogenesis-related protein 5	−2.0124
GRMZM2G075283	Pathogenesis-related protein 1	−1.0306
GRMZM2G112488	Pathogenesis-related protein 10	1.2337
GRMZM2G154449	Pathogenesis-related protein 5	−1.0990
GRMZM2G112524	Pathogenesis-related protein 10	2.2167
**Glutathione S-transferase**		
GRMZM2G146475	Glutathione S-transferase	−0.7240
GRMZM2G161905	Glutathione S-transferase GST 25	2.2247
GRMZM2G129357	Glutathione S-transferase GSTU1	1.1513
GRMZM2G025190	Glutathione S-transferase GSTU6	2.0181
GRMZM2G032856	Glutathione transferase24	−0.7357
GRMZM2G447632	Glutathione S-transferase GSTU6	0.7859
GRMZM2G335618	Glutathione S-transferase GSTU1	2.3108
GRMZM2G028821	Glutathione S-transferase GSTU6	1.7805
GRMZM2G161891	Glutathione transferase35	1.9177
GRMZM2G146913	Glutathione S-transferase GSTU6	4.9274
GRMZM2G064255	Glutathione S-transferase zeta class	0.8050
GRMZM2G052571	Glutathione S-transferase	1.9679
GRMZM2G056388	Glutathione S-transferase GSTU6	1.6273
**Peroxidase**		
GRMZM2G313184	Peroxidase R15	4.6027
AC197758.3_FG004	Peroxidase 52 precursor	1.2116
GRMZM2G135108	Peroxidase	−1.1334
**Heat shock protein**		
GRMZM2G046382	17.4 kDa class I heat shock protein 3	0.7209
**Ferredoxin**		
GRMZM2G048313	Ferredoxin2	−1.0233
**Others**		
GRMZM2G116335	Disease resistance analog PIC16	1.8963
GRMZM2G443525	Disease resistance protein At4g33300-like	0.7199

### Quantitative real-time PCR validation

**Figure 6:**
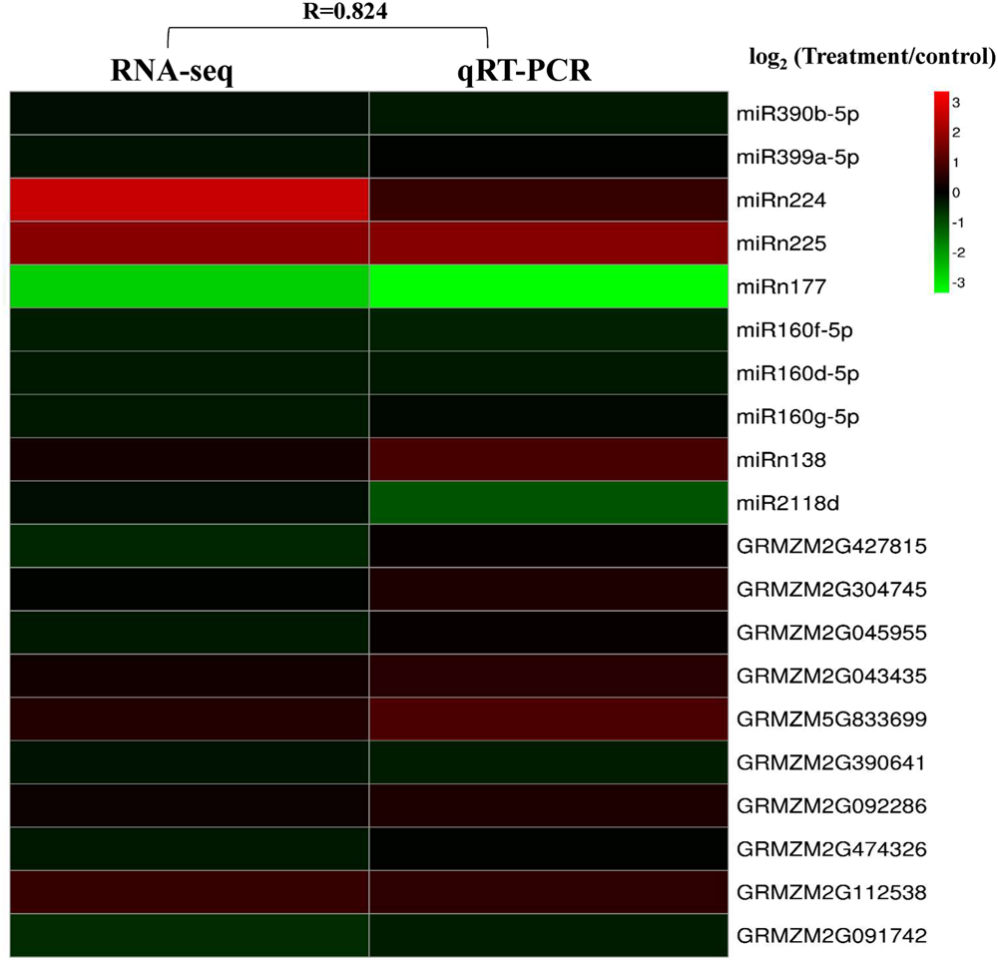
qRT-PCR verification of miRNAs and genes.

To validate the deep-sequencing data, we used quantitative real-time PCR (qRT-PCR) to analyze the expression of miRNAs and mRNAs. Ten miRNAs were selected for qRT-PCR analysis including six known miRNAs and four novel miRNAs. Ten genes were also selected for qRT-PCR analysis. These genes included eight genes related with stress response and two genes related with hormone synthesis and metabolism (Fig. 6, Supplementary Table S10). Pearson correlation values between qRT-PCR and RNA-seq with R = 0.824, suggesting that the sequencing data was consistent with the qRT-PCR results.

## Discussion

### Combined expression analysis of miRNAs and their targets after virus infection

In recent years, high-throughput sequencing method has become a powerful technology for global transcriptome and miRNAome analysis. It has been widely used in many plant species. Here, we simultaneous analyzed the miRNA and gene expression using the same samples before and after RBSDV infection. Through analyzing the relationship between miRNAs and their target genes, twelve miRNA-mRNA pairs were identified, which showed opposing expression patterns response to virus infection (Table 3).

**Table 3:** Potential miRNA/target pairs of maize in response to RBSDV infection.

miRNA family	miRNA name	Target genes	Relative expression level log2 (TL/CL)		Start-end position of target	Scores	Target annotation
			miRNAs	Targets			
**Known miRNAs**							
MiR529	zma-miR529–5p	GRMZM2G160917	4.23	−1.2	1069–1088	2	Squamosa promoter-binding-like protein 12
	zma-miR529–5p	GRMZM2G061734	4.23	−1.7	936–956	2.5	Squamosa promoter-binding-like protein 18
	zma-miR529–5p	GRMZM2G126018	4.23	−3.04	774–794	2.5	Squamosa promoter-binding-like protein 17
MiR528	zma-miR528b-5p	GRMZM2G045955	3.97	−1	1261–1280	2.5	Zea mays rust resistance protein rp3–1
MiR408	zma-miR408b-3p	GRMZM2G331566	4.62	−1.84	174–193	3	Endoglucanase
MiR399	zma-miR399d-5p	GRMZM2G310674	1.98	−1.57	405–425	3	RNA exonuclease 1
MiR398	zma-miR398b-5p	GRMZM2G448151	2.66	−1.87	881–901	3	Small subunit ribosomal protein S3
MiR156	zma-miR156k-5p	GRMZM2G061734	1.64	−1.7	941–961	1	Squamosa promoter-binding-like protein 18
	zma-miR156k-5p	GRMZM2G160917	1.64	−1.2	812–831	1	Squamosa promoter-binding-like protein 14
**Candidate novel miRNAs**							
	zma-miRn53	GRMZM2G076468	1.04	−4.12	632–651	2	Cyclin-P4–1
	zma-miRn138	GRMZM2G160917	1.66	−1.2	814–834	3	Squamosa promoter-binding-like protein 14
	zma-miRn194	GRMZM2G484653	1.15	−1.31	102–121	2	Unknown

**Figure 7:**
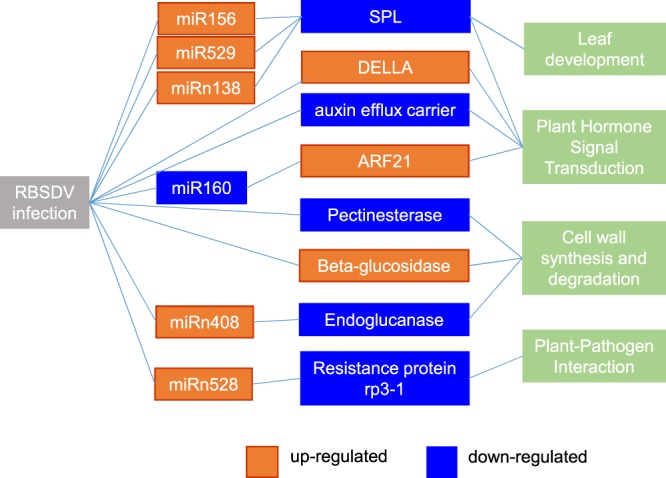
Potential regulatory roles of miRNAs and their targets in maize response to RBSDV.

In rice, miR156/miR529 and *SQUAMOSA PROMOTER BINDING LIKE PROTEIN* (SPL) genes constituted a spatiotemporally coordinated gene network which playing an important regulation roles in tiller and panicle branching^29,30^. Plant SPL genes were involved in leaf development, gibberellin response, light signaling, copper homeostasis, response to stresses, and positively regulate inflorescence meristem^29^. We found miR529 were up-regulated in the virus infected samples, and the expression of its three target genes (SPL genes) were all down-regulated after infected with virus in maize (Table 3, Fig. 7). These results were coincided with the significant phenotype changes of virus infected maize, including the abnormal leaf morphology, dwarf, and the abnormality in vegetative and reproductive growth. One target gene of miR528, which was down-regulated after RBSDV infection, is highly homologous with maize rust resistance protein rp3–1, an important defense gene in maize rust resistance caused by *Puccinia sorghi*^31^. One of the target gene of miR408b-3p is endoglucanase, which catalyzes the hydrolysis of cellulose. Our results showed that miR408b-3p was up-regulated significantly, while its target gene was down-regulated upon virus infection (Table 2, Fig. 7). In addition, the expression trend of miR399d-5p, miR398b-5p and miR156k showed a negative correlation with their target genes. Three novel miRNA-mRNA pairs were identified, of which GRMZM2G076468 encodes a cyclin-dependent protein kinase (Cyclin-P4–1), is the target of zma-miRn53. GRMZM2G160917 is a SPL gene and regulated by zma-miRn138. Zma-miRn194 targeted GRMZM2G484653, a gene annotated as unknown function (Table 2, Fig. 7).

Accumulated evidence indicated that many plant endogenous miRNAs were responsive to pathogenic fungus and virus infection and played important roles in plant disease resistance, such as miR398, miR160^16^, miR482, miR2118^17^, and miR472^18^. After infection with *Magnaporthe Oryzae*, the expression of rice miR398 was induced both in susceptible and resistant lines, while miR160 were only induced in resistant lines, and overexpression of miR160a or miR398b can enhance rice resistance to the disease^16^. Here, we found that the accumulation of miR398 were significantly induced to higher levels upon RBSDV infection, and miR160a decreased upon RBSDV infection (Supplementary Table S3, Fig. 7). The expression trend of miR160 and miR398 in susceptible maize variety B73 was consistent with that in susceptible lines of rice after infected with pathogen. These results suggested miR160 and miR398 might played great roles in maize disease resistance. In addition, miR482, miR2118 and miR472 contribute to plant immunity through negative regulation of R gene^17,18^. However, the expression level of miR2118 was very low, while miR482 and miR472 were not detected in our dataset.

### The expression of genes that involved in dwarf symptoms

After infected with RBSDV, the growth and development of maize exhibited severe abnormalities, such as dwarf, dark green leaf, and failure of completing the reproductive growth in most cases^2,32^. The height of plants is shown to correlate with the composition of the cell wall, which are associated with metabolism and biosynthesis of cellulose, lignin, hemicellulose and pectin^7,33,34^. Cellulose is a major class of polysaccharide, which is the main ingredients of plant cell wall, and was closely relative to plant defense^35^. We identified a variety of gene families involved in cell wall synthesis and degradation, and the expression of these genes altered when the maize infected by RBSDV (Table 4). These genes included 13 cellulose synthase, seven endoglucanase, two glycoside transferase, one glycosyltransferase, five pectin related genes, three glucosidase, galacturonase, one putative mixed-linked glucan synthase and one glycine-rich cell wall structural protein gene (Table 4). Cellulose synthase is an important enzymes important in cellulose synthesis system. In the present study, nine cellulose synthase genes were up-regulated and three cellulose synthase genes were down-regulated upon virus infection. Endoglucanase is a specific enzyme that catalyzes the hydrolysis of cellulose, and the expression of seven endoglucanase encoding genes was altered, five were up-regulated, and two were down-regulated (Table 4). Beta-glucosidase catalyzes the hydrolysis of glycosidic bonds, and a variety of glycosidic conjugates of hormones and defense compounds can be hydrolyzed by beta-glucosidases. Here, we found three beta-glucosidases were up-regulated upon the RBSDV infection. The gene encoding a beta-glucosidases 18 gene was up-regulated for almost thirty times. The up-regulated expression of beta-glucosidases might lead to the degradation of defense compounds, and then resulting in the collapse of maize defense system.

**Table 4:** Expression profile of cell wall synthesis and degradation related genes in response to RBSDV infection.

Gene ID	Annotation	log2(TL/CL)	Expression trend
**Cellulose synthase**			
GRMZM2G111642	cellulose synthase5	0.918326826	up
GRMZM2G018241	cellulose synthase-9	0.964970747	up
GRMZM2G424832	cellulose synthase-4	0.728567596	up
GRMZM2G378836	cellulose synthase A catalytic subunit 6	2.180462068	up
GRMZM2G112336	cellulose synthase A catalytic subunit 10	1.787277899	up
GRMZM2G122431	cellulose synthase-like protein	0.785875406	up
GRMZM2G027723	cellulose synthase A catalytic subunit 2	1.04980425	up
GRMZM2G028353	cellulose synthase-7	1.166988119	up
GRMZM2G025231	cellulose synthase7	1.535744163	up
GRMZM2G339645	cellulose synthase-like	−0.706432842	down
GRMZM2G142898	cellulose synthase A catalytic subunit 7	−1.05281387	down
GRMZM5G876395	cellulose synthase A catalytic subunit 3	−0.971682697	down
GRMZM2G014558	cellulose synthase-like protein E6	−0.739728946	down
**Glucanase**			
GRMZM2G125991	endoglucanase 7	1.288855833	up
GRMZM2G154678	endoglucanase 16	2.481411225	up
GRMZM2G482256	endoglucanase 5	0.73974828	up
GRMZM2G147849	endo-1,4-beta-glucanase Cel1	0.677179814	up
GRMZM2G147849	endo-1,4-beta-glucanase	0.677179814	up
GRMZM2G076348	endo-1,3;1,4-beta-D-glucanase	−0.741829982	down
GRMZM2G331566	endoglucanase	−1.836085972	down
**Glycoside transferase**			
GRMZM2G178025	glycoside transferase	1.034966325	up
AC199765.4_FG008	glycoside transferase	0.971121742	up
**Glycosyltransferase**			
GRMZM2G028286	xyloglucan glycosyltransferase 10	1.213435877	up
**Pectin related**			
GRMZM2G131912	pectate lyase 8	−0.684198742	down
GRMZM2G043415	pectinesterase	−1.700156416	down
GRMZM2G019411	pectinesterase-1	−0.593443161	down
GRMZM2G455564	pectinesterase 8	−0.755441006	down
GRMZM2G012328	pectinesterase inhibitor	−1.107158277	down
**Glucosidase**			
GRMZM2G031628	Beta-glucosidase 18	4.935523594	up
GRMZM2G148176	Beta-glucosidase 8	1.810324677	up
AC234160.1_FG003	Beta-glucosidase 1	1.014596947	up
**Galacturonase**			
GRMZM2G026855	polygalacturonase	0.796011447	up
GRMZM2G333980	polygalacturonase inhibitor 1	0.952508693	up
GRMZM2G004435	polygalacturonase	1.153591577	up
GRMZM2G121312	polygalacturonase inhibitor 2	1.257111081	up
GRMZM2G052844	polygalacturonas	2.586290183	up
GRMZM2G467435	polygalacturonas	0.933885674	up
GRMZM2G002034	polygalacturonas	0.785210875	up
GRMZM2G038281	Beta-galactosidase 3	2.386748111	up
GRMZM2G178106	Beta-galactosidase 5	1.966547727	up
GRMZM2G417455	Beta-galactosidase 3	−1.253589062	down
GRMZM2G127123	Beta-galactosidase 4	−0.841155087	down
GRMZM2G170388	polygalacturonase precursor	−0.726971784	down
GRMZM2G167786	polygalacturonase inhibitor 1	−1.296047742	down
GRMZM2G030265	exopolygalacturonase	−1.09989177	down
GRMZM2G174708	polygalacturonase inhibitor 1 precursor	−2.018772527	down
**Other genes related cell wall structure**			
GRMZM2G103972	putative mixed-linked glucan synthase 1	−1.363662044	down
GRMZM2G109959	glycine-rich cell wall structural protein	2.392335992	up

Plant height is regulated by hormones, such as gibberellins (GAs), auxin (IAA) and brassinosteroid^36–38^. In maize, genes that have large effects on plant height have been well characterized, and most of them were involved in hormone synthesis, transport, and signaling, for example, *brachytic2*^38^, *dwarf3*^39^ and *nana plant1*^40^. In this study, a total of 17 GA biosynthesis and signaling genes were up- or down-regulated upon RBSDV infection, including two DELLA protein genes, four gibberellin 2-beta-dioxygenase genes, seven gibberellin receptor GID1 genes, two gibberellin 20 oxidase (GA20ox) genes, one chitin-inducible gibberellin-responsive gene and one putative response to gibberellin stimulus genes (Table 5). Auxin plays essential roles in regulating plant growth and development, and also regarded as a negative regulator for plant disease resistance^41,42^. In response to RBSDV infection, the expression of many auxin synthesis and transport related genes was alerted. Among them, seven and four auxin responsive factor (ARF) genes were up- and down-regulated upon RBSDV infection, respectively (Table 5). For example, GRMZM2G390641, encoding *ARF21* gene, was regulated by zma-miR160d-5p and miRn91 (Supplementary Table S5-S6).

**Table 5:** Expression profile of gibberellin and auxin related genes in response to RBSDV infection.

Gene ID	Annotation	log2(TL/CL)	Expression trend
**DELLA**			
GRMZM2G001426	DELLA protein	0.880628985	up
GRMZM2G013016	DELLA protein	0.828562365	up
**Gibberellin 2-beta-dioxygenase**			
GRMZM2G152354	gibberellin 2-beta-dioxygenase	1.370160759	up
GRMZM2G031432	gibberellin 2-beta-dioxygenase	2.637072082	up
GRMZM2G051619	gibberellin 2-beta-dioxygenase	3.010518964	up
GRMZM2G006964	gibberellin 2-beta-dioxygenase 8	−1.351721572	down
**Gibberellin receptor GID1**			
GRMZM2G301934	gibberellin receptor GID1L2	1.559501317	up
GRMZM2G420786	gibberellin receptor GID1L2	0.693598092	up
GRMZM2G111421	gibberellin receptor GID1L2	0.701789901	up
GRMZM2G173630	GID1-like gibberellin receptor	0.740894975	up
GRMZM2G016605	gibberellin receptor GID1	1.446037371	up
GRMZM2G049675	gibberellin receptor GID1L2	0.860587685	up
GRMZM5G831102	gibberellin receptor GID1L2 precursor	−0.623988411	down
**Gibberellin 20 oxidase**			
GRMZM2G050234	gibberellin 20 oxidase	2.41338231	up
AC203966.5_FG005	gibberellin 20 oxidase 1	1.916779027	up
**Other genes related gibberellin**			
GRMZM2G098517	chitin-inducible gibberellin-responsive	0.705234195	up
AC205471.4_FG007	unknown (response to gibberellin stimulus)	3.204932625	up
**Auxin response factor (ARF)**			
GRMZM2G028980	auxin response factor 16 (ARF16) gene	1.151955362	up
GRMZM2G081158	auxin response factor 9 (ARF9) gene	1.65654925	up
GRMZM2G153233	auxin response factor 2 (ARF2) gene	1.413686542	up
GRMZM2G073750	auxin response factor 9 (ARF9) gene	0.732854439	up
GRMZM2G703565	auxin response factor 5 (ARF5) gene	1.991605559	up
GRMZM2G035405	auxin response factor 18 (ARF18) gene	1.186726815	up
GRMZM2G078274	auxin response factor 3 (ARF3) gene	0.743864138	up
GRMZM2G034840	auxin response factor 4 (ARF4) gene	−1.391198729	down
GRMZM2G390641	auxin response factor 21 (ARF21) gene	−0.790919233	down
GRMZM2G137413	auxin response factor 14 (ARF14) gene	−0.720913025	down
GRMZM2G437460	auxin response factor 12 (ARF12) gene	−0.975080801	down
**Other auxin response genes**			
GRMZM2G154332	SAUR12-auxin-responsive	1.109550229	up
GRMZM2G057067	IAA6-auxin-responsive	0.961062035	up
GRMZM2G138268	auxin-responsive protein	0.89357466	up
GRMZM5G864847	IAA16-auxin-responsive	0.784317249	up
GRMZM5G835903	SAUR55-auxin-responsive	−0.84926364	down
GRMZM2G343351	SAUR44-auxin-responsive	−0.882790627	down
GRMZM2G465383	SAUR25-auxin-responsive	−1.212035079	down
**IAA synthesis**			
GRMZM2G053338	Indole-3-acetic acid amido synthetase (GH3)	3.923877451	
**Auxin transporter-like protein**			
GRMZM2G126260	auxin efflux carrier PIN10a (PIN10a)	1.74186011	up
GRMZM2G025742	auxin efflux carrier component 6	−1.948711258	down
GRMZM2G098643	auxin efflux carrier	−0.830424288	down
GRMZM2G382393	auxin Efflux Carrier family protein	−1.373387099	down
GRMZM2G171702	auxin efflux carrier PIN1d (PIN1d) gene	−0.772559276	down
GRMZM2G025659	auxin efflux carrier PIN5a (PIN5a) gene	−1.188365531	down
GRMZM2G175983	auxin efflux carrier PIN5a (PIN5a) gene	−2.788212274	down
GRMZM2G149481	auxin transporter-like protein 3	−2.689255972	down

Auxin polar transport is essential for the formation and maintenance of local auxin gradients of plant^43,44^. Auxin efflux carriers PIN family genes played important roles in auxin polar transport. Loss of function of PIN genes severely affected organ initiation. For example, the auxin transport-defective mutants *br2* and *sem1* showed dwarf phenotype and vasculature defects^43^. Here, the expression level of seven auxin efflux carrier genes were decreased after virus infection (Table 5). It is possible that the decreasing expression of auxin efflux carrier protein genes could be a major reason that caused the dwarf phenotype after maize infected by RBSDV.

In conclusion, we identified 302 miRNAs and 351 potential target genes in maize. The expression patterns of 81 miRNAs differed dramatically upon RBSDV infection. Combined small RNA and gene expression analysis identified 12 miRNA-mRNA pairs with opposite expression patterns response to virus infection, and six of them are likely to play significant roles in the formation of maize disease symptoms. This study provided insight into the roles of miRNAs in response to RBSDV, and could help to develop novel strategy for crops against virus infection.

## Materials and Methods

*Zea mays* B73 was planted in the field where the RBSDV disease happened seriously almost every year. As the control, the plants were covered with a net to prevent the planthoppers. Leaves of one-month-old maize seedlings with rough dwarf disease symptoms were collected. Total RNA were prepared separated from each individual sample using RNAiso Plus reagent (Takara, Dalian, China), following by RNase-free DNase treatment (Takara, Dalian, China). RNA concentration was quantified by Eppendorf BioPhotometer plus UV-Visible Spectrophotometer. The cDNA were synthesis using One Step PrimeScript miRNA cDNA Synthesis Kit (Takara, Dalian, China) according the manufacturer’s instructions. According to the sequences of RBSDV segment S6 and S7 (HF955010, HF955011), two pair of primers pS6–604 (5′-CCTAGTTCTCCGCAAGCC-3′, 5′-CAGGGACAGTTCCAATCATAAA-3′) and pS7–342 (5′- TCAGCAAAAGGTAAAGGAAGG -3′, 5′- GCTCCTACTGAGTTGCCTGTC-3′) were designed. Samples were collected according the method in previous studies^7,45^. Every ten virus infected seedlings which can be detected by both the primer pS6–604 and pS7–342 were harvested as one sample, and three more samples were prepared by the same method as replicates.

### Construction and sequencing of small RNA libraries

Small RNA libraries were constructed as described in the previous studies^15,46^. Briefly, low molecular weight RNAs (10 nt - 40 nt) were isolated from the total RNA by electrophoresis using 15% TBE-urea denaturing polyacrylamide gel. Then, the 5′ and 3′ adaptors were added and reverse transcription was performed to synthesize cDNA. And cDNA libraries were sequenced using Illumina HiSeqTM 2000. The sequencing was accomplished by BGI small RNA pipeline (BGI, Shenzhen, China). After sequencing, clean reads were generated by removing the adapter sequences and low quantity reads (reads having ‘N’, <18 nt, and >29 nt). Then clean reads were used to align with maize B73 RefGen_V2 genome (http://archive.maizesequence.org/index.html), GenBank and Rfam database, and miRbase (http://www.mirbase.org/). The detail processes to identify known and novel miRNAs were according to the method described in previous studies^47^.

### Transcriptome sequencing and bioinformatics analysis

Transcriptome libraries were constructed using Illumina sample preparation kits. Briefly, poly A mRNAs were isolated and cut to short fragments. The short mRNA fragments were then used to synthesize the first strand cDNA using random hexamers primers. Then, dNTPs, RNase H, buffer and DNA polymerase I were added to synthesize the second strand cDNA. cDNAs were further purified using QiaQuick PCR kit. Then, polyA tails and adaptors were added and the DNA fragments with suitable size were recovered from gel. Finally, the cDNA were amplified by PCR, followed by sequencing using Illumina HiSeq™ 2000.

After sequencing, raw data was filtered to generate clean reads by removing adaptor sequences, reads containing multiple N and lower quality reads. Then, the clean reads were used to compare with maize genome sequences (B73 RefGen_V2, release 5b.60) using SOAPaligner/SOAP2 with the parameters that mismatch ≤2^48^. The gene expression level is calculated by using FPKM method^49^. Differential expression analysis of two samples was performed using rigorous algorithm method with P-value ≤ 0.001 and the absolute value of log2Ratio ≥1. Gene function analysis was carried out by BLASTx searches against the UniProt database and the Swiss-Prot protein database (http://www.expasy.ch/ sprot). Gene Ontology (GO) annotation analysis was based on WEGO (http://wego.genomics.org.cn/cgi-bin/wego/index.pl). Cluster of Orthologous Groups (COG) classification analysis was based on the database (http://www.ncbi.nlm.nih.gov/COG/). Pathway-based analysis was performed according to Kyoto Encyclopedia of Genes and Genomes Pathway (KEGG) database (http://www.genome.jp/kegg/).

### qRT-PCR Validation

For qRT-PCR validation of miRNAs, the Mir-X miRNA qRT-PCR SYBR Kit (Clontech Laboratories, Inc) were used following the manufacturer’s instructions. For all miRNAs and genes, the qRT-PCR was performed in ABI7500 (Applied Biosystems). Primers used for qRT-PCR were listed in Supplementary Table S11. All reactions were performed in biological triplicates. For qRT-PCR analysis of miRNAs and mRNAs, U6 RNA and ubiquitin were used as the internal control, respectively. The relative expression of all mRNAs and miRNAs were calculated using 2^−ΔΔct^ method.

## Electronic supplementary material

Supplemental information

Dataset 1

Dataset 2

Dataset 3

Dataset 4

Dataset 5

Dataset 6

Dataset 7

Dataset 8

Dataset 9

Dataset 10

Dataset 11
