# Supplementary material for: Combined small RNA and gene expression analysis revealed roles of miRNAs in maize response to rice black-streaked dwarf virus infection
Source: Sci Rep. 2018 Sep 10;8:13502. doi: 10.1038/s41598-018-31919-z (PMC6131507; doi:10.1038/s41598-018-31919-z)
Supplement: Supplementary file 1 — Supplemental information [file 41598_2018_31919_MOESM1_ESM.pdf]

Title：Combined small RNA and gene expression analysis revealed roles of miRNAs in maize response to rice black-streaked dwarf virus infection

Authors：Aiqin Li[1], Guanghui Li [1], Yuhan Zhao[2], Zhaodong Meng[3], Meng Zhao[3], Changsheng Li[1], Ye Zhang[1], Pengcheng Li[1,2], Chang-Le Ma[3], Han Xia[1,2], Shuzhen Zhao[1,2], Lei Hou[1], Chuanzhi Zhao[1,2]*, Xingjun Wang[1,2]*

Aiqin Li and Guanghui Li contributed equally to this work.

Affiliations:

[1] Biotechnology Research Center, Shandong Academy of Agricultural Sciences; Shandong Provincial Key Laboratory of Crop Genetic Improvement, Ecology and Physiology, Jinan 250100, PR China.

[2] College of Life Sciences, Shandong Normal University, Jinan 250014, PR China

[3] Maize Research Institute, Shandong Academy of Agricultural Sciences, Jinan 250100, PR China.

*** Corresponding author**

Xingjun Wang, Email: xingjunw@hotmail.com

Chuanzhi Zhao, Email: chuanzhiz@126.com

Supplementary Data

Table S1. Summary of small RNA reads from four libraries

Table S2. Classification of clean reads generated from four small RNA libraries

Table S3. Detail information of know miRNAs identified

Table S4. Detail information of novel miRNAs identified

Table S5. Target genes of known miRNAs

Table S6. Target genes of novel miRNAs

Table S7. Different expressed miRNAs identified from small RNA libraries

Table S8. Summary of RNA-seq reads from the individual small RNA library

Table S9. Function annotation of differentially expressed genes

Table S10. Validation of RNA-seq results via qRT-PCR.

Table S11. Primers for qRT-PCR analysis.

Supplementary Information

Figure S1. The morphology and molecular detection of RBSDV infected plants. a: Non-infected control plants. b: The RBSDV infected maize seedlings. c: Molecular detection of RBSDV using virus specific primers. T1-T5 were the individual plants infected by RBSDV.

Figure S2. Differentially expressed novel miRNAs identified from small RNA libraries

Figure S3. Sequencing randomness and saturation analysis for transcriptome sequencing

Figure S4. COG classification analysis of differentially expressed genes

Figure S5. GO annotation of differentially expressed genes

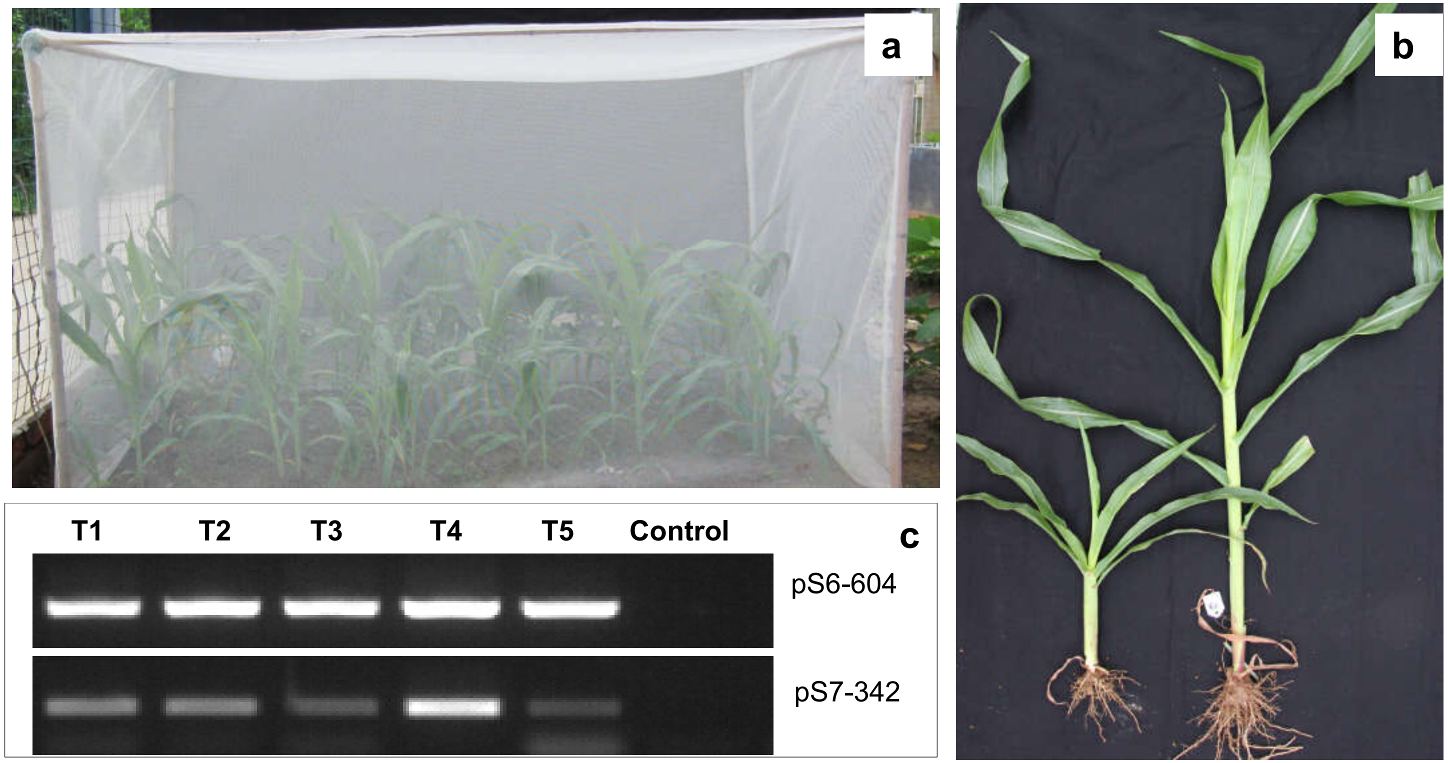

Figure S1

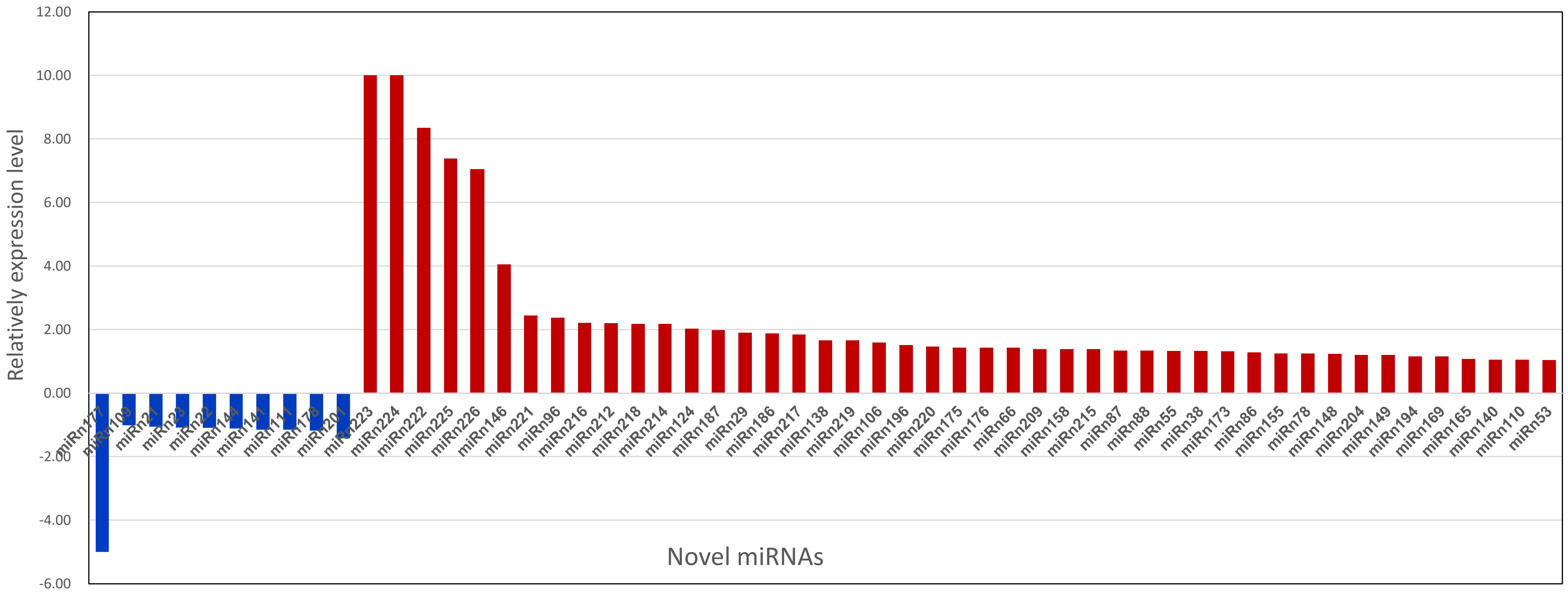
Relatively expression level
12.00
10.00
8.00
6.00
4.00
2.00
0.00
-2.00
-4.00
-6.00
Novel miRNAs
miRn177
miRn109
miRn21
miRn23
miRn22
miRn144
miRn141
miRn111
miRn178
miRn201
miRn223
miRn224
miRn222
miRn225
miRn226
miRn146
miRn221
miRn96
miRn216
miRn212
miRn218
miRn214
miRn124
miRn187
miRn29
miRn186
miRn217
miRn138
miRn219
miRn106
miRn196
miRn220
miRn175
miRn176
miRn66
miRn209
miRn158
miRn215
miRn87
miRn88
miRn55
miRn38
miRn173
miRn86
miRn155
miRn78
miRn148
miRn204
miRn149
miRn194
miRn169
miRn165
miRn140
miRn110
miRn53

Figure S2

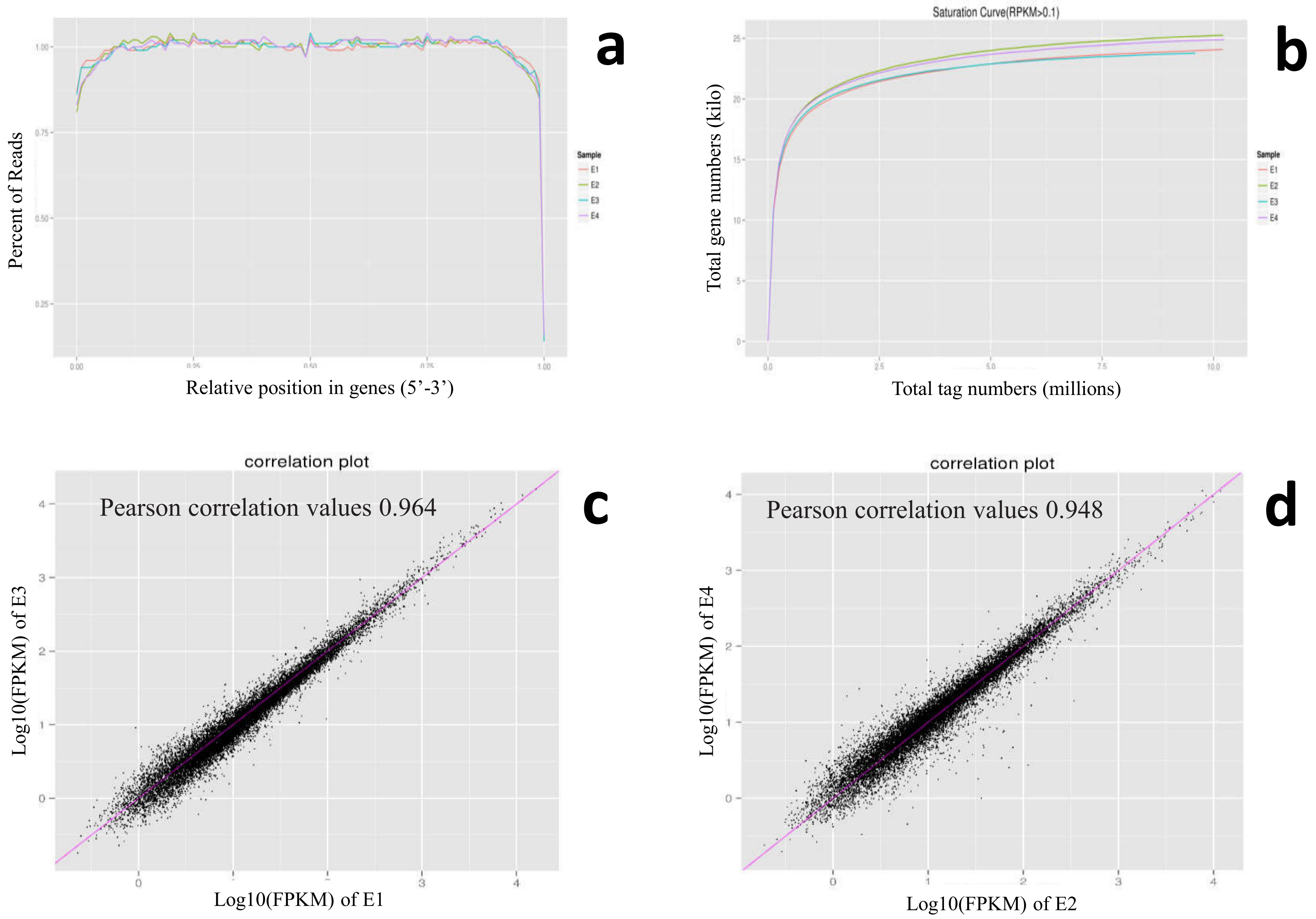
a
Percent of Reads
Relative position in genes (5’-3’)
Sample
E1
E2
E3
E4
b
Saturation Curve(RPKM>0.1)
Total gene numbers (kilo)
Total tag numbers (millions)
c
correlation plot
Pearson correlation values 0.964
Log10(FPKM) of E3
Log10(FPKM) of E1
d
correlation plot
Pearson correlation values 0.948
Log10(FPKM) of E4
Log10(FPKM) of E2

Figure S3

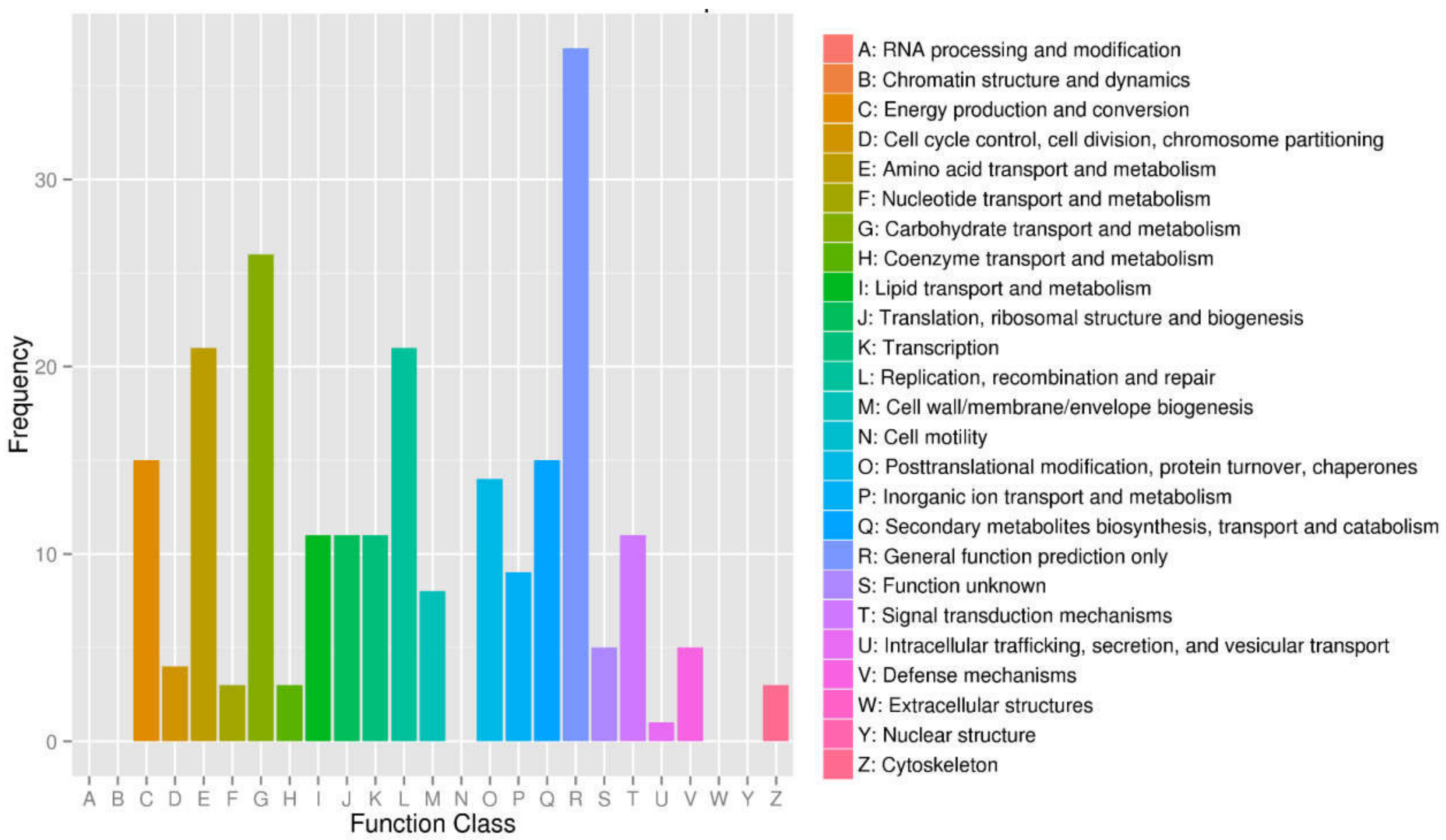
Frequency
30
20
10
0
A B C D E F G H I J K L M N O P Q R S T U V W Y Z
Function Class
A: RNA processing and modification
B: Chromatin structure and dynamics
C: Energy production and conversion
D: Cell cycle control, cell division, chromosome partitioning
E: Amino acid transport and metabolism
F: Nucleotide transport and metabolism
G: Carbohydrate transport and metabolism
H: Coenzyme transport and metabolism
I: Lipid transport and metabolism
J: Translation, ribosomal structure and biogenesis
K: Transcription
L: Replication, recombination and repair
M: Cell wall/membrane/envelope biogenesis
N: Cell motility
O: Posttranslational modification, protein turnover, chaperones
P: Inorganic ion transport and metabolism
Q: Secondary metabolites biosynthesis, transport and catabolism
R: General function prediction only
S: Function unknown
T: Signal transduction mechanisms
U: Intracellular trafficking, secretion, and vesicular transport
V: Defense mechanisms
W: Extracellular structures
Y: Nuclear structure
Z: Cytoskeleton

Figure S4

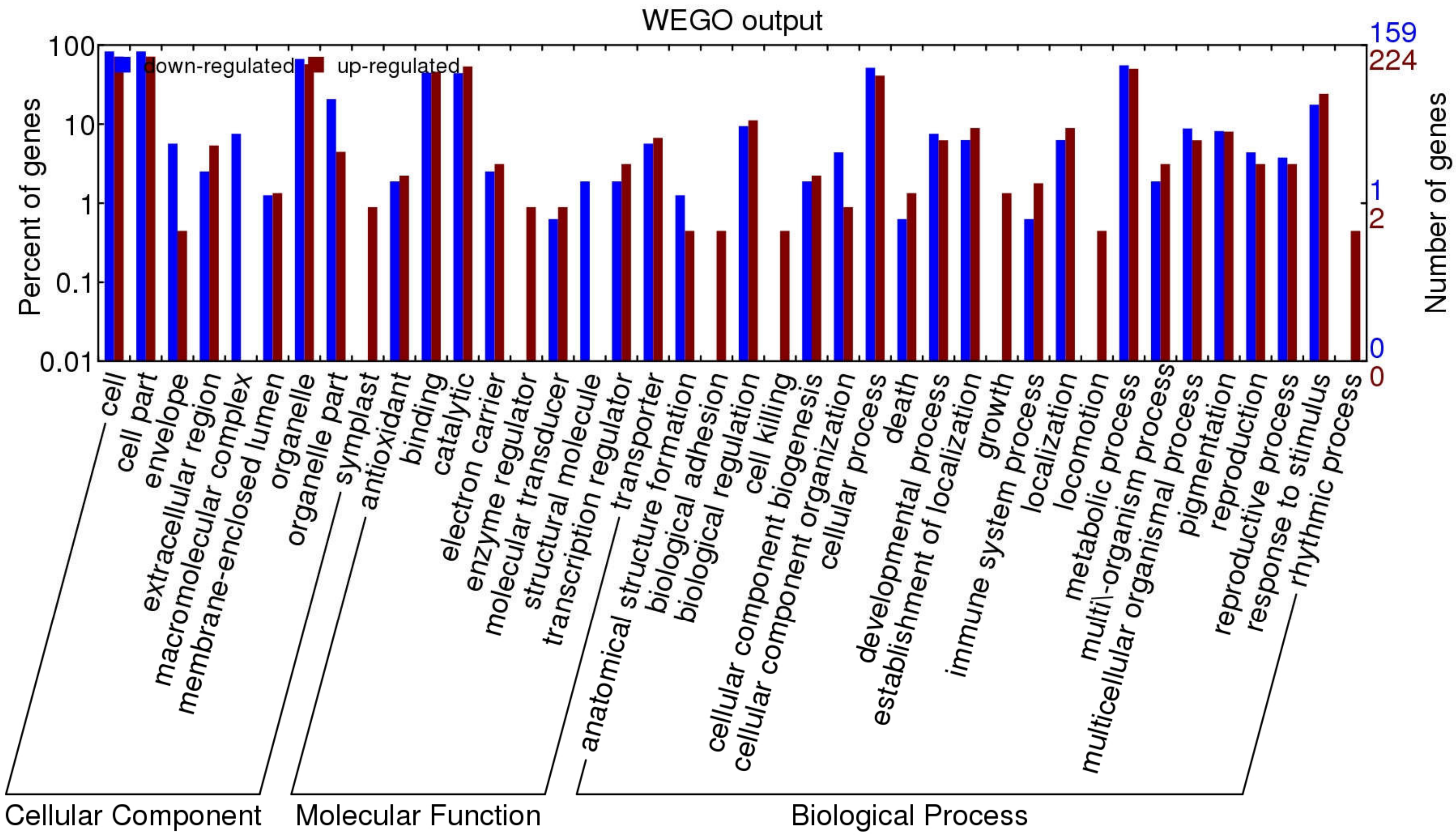
WEGO output
down-regulated
up-regulated
Percent of genes
Number of genes
100
10
1
0.1
0.01
159
224
1
2
0
0
cell
cell part
envelope
extracellular region
macromolecular complex
membrane-enclosed lumen
organelle
organelle part
symplast
antioxidant
binding
catalytic
electron carrier
enzyme regulator
molecular transducer
structural molecule
transcription regulator
transporter
anatomical structure formation
biological adhesion
biological regulation
cell killing
cellular component biogenesis
cellular component organization
cellular process
death
developmental process
establishment of localization
growth
immune system process
localization
locomotion
metabolic process
multi\-organism process
multicellular organismal process
pigmentation
reproduction
reproductive process
response to stimulus
rhythmic process
Cellular Component
Molecular Function
Biological Process

Figure S5
